# Bone turnover markers (β-CTX, PINP, ALP) in osteoporosis: correlation with bone loss and fracture risk stratification

**DOI:** 10.3389/fendo.2025.1628434

**Published:** 2026-01-05

**Authors:** Shiqiang Wu, Liangming Wang, Xiaolu Zhang, Liquan Cai, Qingfeng Ke, Jie Xu

**Affiliations:** 1 Department of Orthopedic, The Second Affiliated Hospital of Fujian Medical University, Quanzhou, China; 2 Shengli Clinical Medical College of Fujian Medical University, Fuzhou, Fujian, China

**Keywords:** bone turnover markers, β-C-terminal telopeptide of type I collagen (β-CTX), alkaline phosphatase, procollagen type I N-terminal propeptide (PINP), osteoporosis, fracture

## Abstract

**Objective:**

To investigate the correlation of β-C-terminal telopeptide of type I collagen (β-CTX), procollagen type I N-terminal propeptide (PINP), alkaline phosphatase (ALP) with bone mineral density (BMD) in patients with osteoporosis and evaluate their predictive value for secondary fracture risk.

**Methods:**

A total of 180 osteoporosis patients and 80 healthy controls were enrolled. The osteoporosis group was stratified into fracture and non-fracture cohorts. Correlation of β-CTX, PINP, ALP with BMD at lumbar spine (L1-4) and femoral neck was assessed using Pearson correlation coefficient. Binary logistic regression model was employed to analyze the risk factors associated with secondary fractures in patients with osteoporosis and receiver-operator characteristic (ROC) curve was used for assessing the predictive utility.

**Results:**

Osteoporosis patients exhibited significantly higher β-CTX, PINP, and ALP levels compared to controls (all *P* < 0.001), alongside lower BMD at both lumbar spine and femoral neck (*P* < 0.001). Negative correlations were observed between β-CTX, PINP, ALP, and BMD (r = -0.553 to -0.671, P < 0.001). The fracture group (n=83 vs. n=97 non-fracture) revealed elevated β-CTX, PINP, and ALP levels (all *P* < 0.001). Logistic regression identified β-CTX, PINP, and ALP as independent risk factors. ROC analysis demonstrated strong predictive accuracy for combined β-CTX, PINP, ALP and BMD (AUC = 0.943), outperforming individual parameters.

**Conclusion:**

Elevated β-CTX, PINP, and ALP levels correlate with reduced BMD and independently predict secondary fracture risk in osteoporosis patients. Integrating these biomarkers with BMD enhances fracture risk stratification, supporting their inclusion in clinical risk assessment tool.

## 1 Introduction

Osteoporosis, a prevalent metabolic bone disease characterized by reduced bone mass and microarchitectural deterioration, leads to increased bone fragility and fracture risk (1, 2). It poses a substantial global health burden, with fractures affecting approximately one in three women and one in five men over the age of 50 (3, 4), resulting in significant morbidity and healthcare costs. This underscores the critical need for improved risk stratification tools.

While bone mineral density (BMD) remains a cornerstone for osteoporosis diagnosis and fracture risk assessment, accumulating evidence highlights its limitations. It has been reported that only about 30% to 50% of patients with osteoporosis fracture showed symptoms of reduced BMD (5), suggesting that relying solely on BMD may not provide complete picture of fracture risk in patients with osteoporosis and underscoring the need for complementary biomarkers to refine risk stratification.

Consequently, bone turnover markers (BTMs), which reflect the dynamic process of bone remodeling, have garnered interest as complementary biomarkers (5). Specifically, β-C-terminal telopeptide of type I collagen (β-CTX) and procollagen type I N-terminal propeptide (PINP) are recommended as reference markers by the International Osteoporosis Foundation (IOF) for assessing resorption and formation, respectively (6) Alkaline phosphatase (ALP) is also a widely available marker of osteoblastic activity (7).

However, several knowledge gaps persist. Most evidence derives from studies on postmenopausal women, leaving their utility in mixed-gender cohorts underexplored. Moreover, the combined predictive value of key BTMs (β-CTX, PINP, and ALP) alongside BMD has not been well established, and these markers are not integrated into widely used tools like FRAX (5). Therefore, the present study aimed to: (1) compare levels of β-CTX, PINP, ALP, and BMD between osteoporosis patients and healthy controls; (2) analyze their correlations within a mixed-gender cohort; and (3) evaluate their individual and combined utility for stratifying secondary fracture risk.

## 2 Materials and methods

### 2.1 Study subjects

We conducted an observational study comparing bone turnover markers and BMD between patients diagnosed with osteoporosis and healthy individuals at the Second Affiliated Hospital of XX Medical University. Following screening of inclusion and exclusion criteria, a total of 180 osteoporosis patients who received treatment at our hospital from January 2021 to December 2022 were recruited, and 80 age- and BMI-matched healthy controls who underwent physical examinations during the same timeframe were selected.

Sample size calculation was performed using G*Power 3.1 software (α= 0.05, β= 0.20, effect size= 0.40 based on study design and prior studies (8)). The effect size was chosen based on comparable studies investigating the correlation between BTMs and BMD, ensuring adequate power to detect clinically significant differences.

Inclusion criteria were as follows. For the osteoporosis group, participants met diagnostic criteria for osteoporosis: T-score ≤ -2.5 standard deviations (SD) at lumbar spine [L1-4] or femoral neck via dual-energy X-ray absorptiometry (DEXA); patients had not received osteoporosis-related medications prior to enrollment. For the control group, individuals had no history of orthopedic diseases and exhibited normal bone density. All study subjects were mentally competent and voluntarily participated, having provided informed consent.

Exclusion criteria were: (1) abnormal thyroid and liver function; (2) chronic kidney disease (CKD) defined by chronicestimated glomerular filtration rate (eGFR) <60 mL/min/1.73 m^2^; (3) malignancy or autoimmune diseases; (4) diabetes or hypertension; (5) history of fragility fractures or other fractures occurred at vertebra, hip or wrist; (6) secondary osteoporosis (e.g., due to glucocorticoid use, hyperthyroidism); (7) use of medications affecting bone metabolism in the past six months; (8) metabolic bone disorders (e.g., Paget’s disease).

### 2.2 Measurement of β-CTX, PINP, and ALP

Fasting venous blood samples (3mL) were collected from all participants in the morning and serum was isolated by centrifugation at 5000 r/min for 15 minutes and stored at -80 °C. Serum levels of β-CTX and intact PINP levels were quantified using electrochemiluminescence immunoassays (Roche Diagnostics, Germany; intra-/inter-assay CV < 5%), with results expressed in ng/mL. Total ALP activity was measured using a p-nitrophenyl phosphate (pNPP) kinetic assay (Roche Diagnostics, Germany) according to manufacturer protocols, with inter-assay CV <3%. For quality control, serum samples and calibrators were processed in duplicate. In the ALP assay, 10 μL of serum was incubated with 1 mL pNPP substrate solution (37 °C, 5 min). Enzyme activity was determined by monitoring the absorbance change at 405 nm over 3 minutes using a Hitachi 7600 automated analyzer (Hitachi High-Tech, Japan), with results expressed in U/L.

### 2.3 Measurement of BMD

BMD measurements of the lumbar spine (L1-4) and femoral neck were obtained using the Discovery Wi-type X-ray BMD detector from Hologic for all study subjects (Discovery Wi DXA system, Hologic, Bedford, MA), with results expressed in grams per cm^2^.

### 2.4 Evaluation of secondary fracture

Participants with osteoporosis were stratified into fracture and non-fracture cohorts based on radiographic evidence of fragility fractures. The fracture group included cases with low-trauma fractures (e.g., falls from standing height or less) at typical osteoporotic sites (hip, spine, or wrist). Cases with pathological fractures caused by malignancies or high-energy trauma were excluded. The non-fracture group consisted of participants without radiographic evidence of prior fragility fractures and no history of traumatic fractures.

### 2.5 Outcome measures

(1) Comparison of serum levels of β-CTX, PINP, and ALP between the osteoporosis and healthy groups, as well as their correlation with lumbar spine (L1-4) and femoral neck BMD; (2) comparison of β-CTX, PINP, and ALP levels and BMD between the fracture and non-fracture groups; (3) binary logistic regression of risk factors for secondary fractures in osteoporosis patients; (4) receiver operating characteristic (ROC) curve analysis of serum β-CTX, PINP, ALP, and BMD for predicting secondary fractures in patients with osteoporosis.

### 2.6 Statistical analysis

Data were analyzed using SPSS 22.0 (IBM, USA). Normality was assessed via Kolmogorov-Smirnov test. Mean (standard deviation [SD]) and median (interquartile range [IQR]) were used for description of normally and non-normally distributed continuous data, respectively. Group comparisons employed independent t-tests or Mann-Whitney U tests. Categorical variables were analyzed using the chi-square test. The relationship between serum β-CTX, PINP, ALP and BMD was analyzed by Pearson correlation coefficient and binary logistic regression model was employed to analyze the risk factors associated with secondary fractures in patients with osteoporosis. Additionally, ROC curve analysis was conducted to investigate the predictive efficacy of β-CTX, PINP, ALP and BMD for secondary fractures in patients with osteoporosis. Logistic regression model fitness was assessed using the Hosmer-Lemeshow test, and multicollinearity among independent variables was evaluated by variance inflation factor (VIF). A *P* < 0.05 indicates statistical significant differences in data comparisons.

## 3 Results

### 3.1 Comparisons between the osteoporosis group and healthy control group

In the osteoporosis group, there were 88 male and 92 female (51.1%) participants, with mean age of 76.85 (± 5.42) and mean body mass index (BMI) of 24.11 (± 2.13) kg/m^2^. In the healthy group, there were 41 male and 39 female (48.8%) participants, with mean age of 77.02 (± 5.56) and mean BMI of 24.24 (± 2.15) kg/m^2^. The two groups were comparable with no significant differences found (*P* > 0.05).

As shown in **Table 1**, serum levels of β-CTX, PINP, and ALP were significantly elevated in the osteoporosis group compared to the healthy group (*P* < 0.001 for all markers). Regarding BMD, the BMD values at the lumbar spine (L_1-4_) and femoral neck were markedly lower in the osteoporosis group, as compared to the healthy group (*P* < 0.001 for both sites) (**Table 2**).

**Table 1:** Comparison of serum β-CTX, PINP, and ALP levels between the osteoporosis and control groups.

Items	β-CTX (ng/mL)	PINP (ng/mL)	ALP (U/L)
Osteoporosis group (n=180)	0.78 ± 0.13	68.85 ± 6.79	86.42 ± 10.14
Control group (n=80)	0.16 ± 0.05	30.12 ± 2.43	54.54 ± 5.26
*t*	41.290	49.580	26.560
*P*	<0.001	<0.001	<0.001

Data were expressed as mean ± SD. ALP, alkaline phosphatase; β-CTX, β-C-terminal telopeptide of type I collagen; PINP, procollagen type I N-terminal propeptide.

**Table 2:** Comparison of bone mineral density between the osteoporosis and control groups.

Items	Lumbar vertebra L_1~4_ BMD (g/cm^2^)	Femoral neck BMD (g/cm^2^)
Osteoporosis group (n=180)	0.40 ± 0.05	0.35 ± 0.03
Control group (n=80)	0.98 ± 0.12	1.12 ± 0.19
*t*	55.070	53.030
*P*	<0.001	<0.001

Data were expressed as mean ± SD. BMD: bone mineral density.

### 3.2 Correlation of serum β-CTX, PINP, and ALP with BMD in patients with osteoporosis

**Figure 1:**
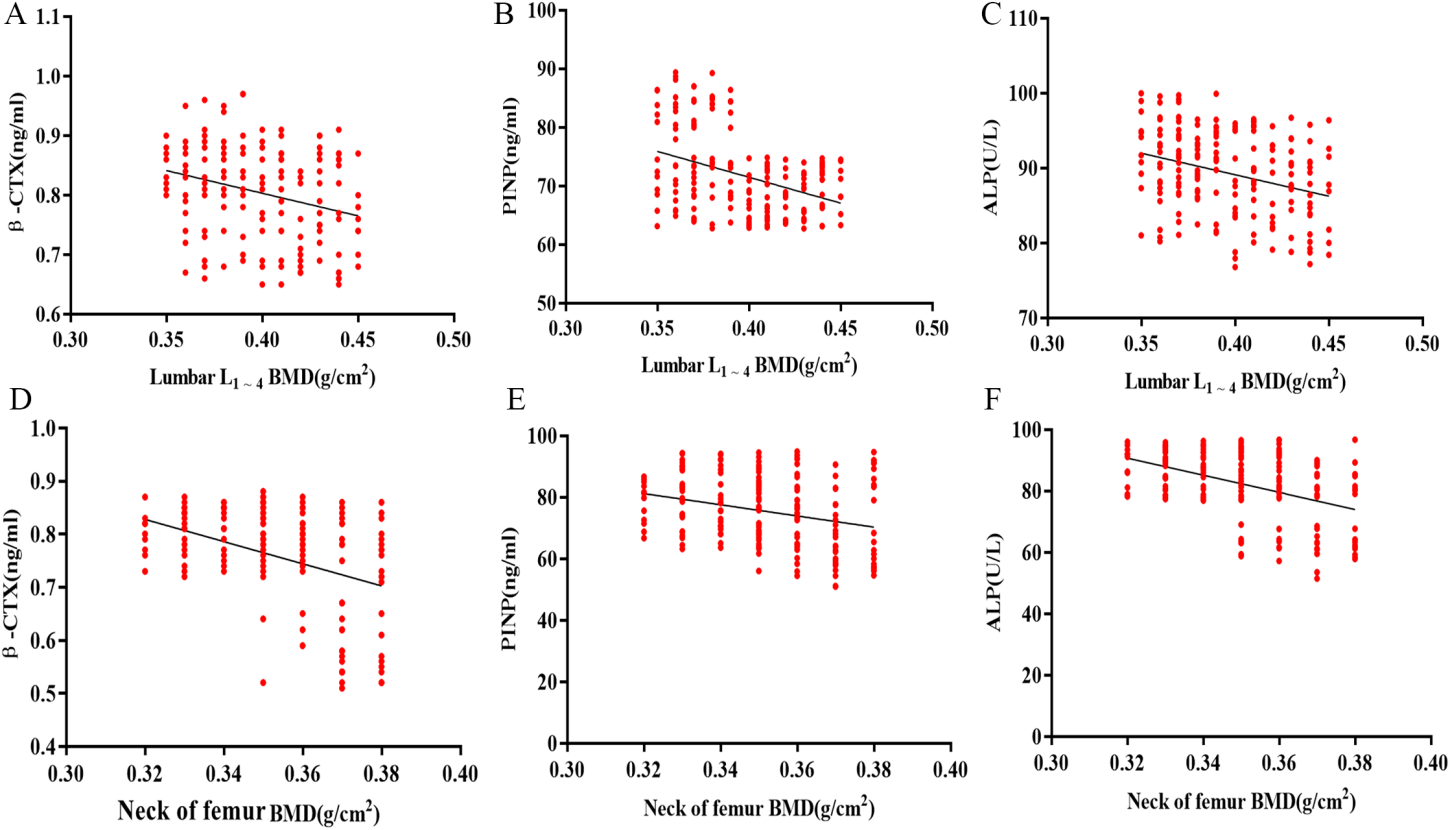
Scatter plots illustrating the correlations between serum bone turnover markers and BMD in osteoporosis patients. **(A)** β-CTX vs. lumbar spine (L1–4) BMD; **(B)** PINP vs. lumbar spine (L1–4) BMD; **(C)** ALP vs. lumbar spine (L1–4) BMD; **(D)** β-CTX vs. femoral neck BMD; **(E)** PINP vs. femoral neck BMD; **(F)** ALP vs. femoral neck BMD. ALP: alkaline phosphatase; β-CTX: β-C-terminal telopeptide of type I collagen; PINP: procollagen type I N-terminal propeptide.

Negative correlations were observed between these serum bone turnover markers and BMD in osteoporosis patients. β-CTX, PINP, and ALP exhibited significant inverse relationships with lumbar spine BMD (r = -0.553, -0.573, and -0.612; *P* < 0.001) and femoral neck BMD (r = -0.671, -0.559, and -0.598; *P* < 0.001). See **Table 3** and **Figure 1**.

**Table 3:** Correlation coefficients of BMD with serum β -CTX, PINP and ALP.

Items	β-CTX	PINP	ALP
Lumbar vertebra L_1~4_ BMD	r =-0.553, P <0.001	r =-0.573, P <0.001	r =-0.612, P <0.001
Femoral neck BMD	r =-0.671, P <0.001	r =-0.559, P <0.001	r =-0.598, P <0.001

ALP, alkaline phosphatase; β-CTX: BMD, bone mineral density; β-CTX, β-C-terminal telopeptide of type I collagen; PINP, procollagen type I N-terminal propeptide.

### 3.3 Comparison between fracture groups and non-fracture groups

Osteoporosis patients were subsequently stratified into fracture (n = 83) and non-fracture (n = 97) subgroups. The comparisons of clinical profile between the two groups are summarized in **Table 4**. As shown, significant differences were noted in age distribution (≥ 60 years: 63.86% vs. 42.27%, *P* < 0.001), occupational type (manual labor: 71.08% vs. 15.46%, *P* < 0.001), and calcium/vitamin D supplementation (39.76% vs. 70.10%, *P* < 0.001). Additionally, the fracture group had lower serum levels of hemoglobin, albumin, uric acid, and creatinine, relative to the non-fracture group (*P* < 0.001). **Supplementary Table S1** summarizes the comparison of key lifestyle and nutritional variables, revealing that the fracture group had significantly higher rates of smoking and alcohol consumption, lower dietary calcium intake, and a greater proportion of individuals engaged in high-level physical activity compared to the non-fracture group.

**Table 4:** Comparison of the clinical data between the fracture group and the non-fracture group.

Clinical data		Fracture group (n =83)	Non-fracture group (n =97)	*χ*^2^/t	*P*
Gender	Male	42 (50.60)	46 (47.42)	0.181	0.671
	Female	41 (49.40)	51 (52.58)		
Age (year)	≥60	53 (63.86)	41 (42.27)	8.354	<0.001
	<60	30 (36.14)	56 (57.73)		
BMI (kg/m^2^)		24.35 ± 2.19	24.31 ± 2.31	0.119	0.906
Occupational type	Manual labour	59 (71.08)	15 (15.46)	57.710	<0.001
	Brain work	15 (18.07)	59 (60.82)		
	Other	9 (10.84)	23 (23.71)		
Use of calcium agent/ vitamin D	Yes	33 (39.76)	68 (70.10)	16.723	<0.001
	No	50 (60.24)	29 (29.90)		
Osteoporosis Treatment	Surgical operation	48 (57.83)	57 (58.76)	0.016	0.899
	Anti-osteoporosis drugs	35 (42.17)	40 (41.24)		
Blood Biochemical Indicators	Hemoglobin (g / L)	114.53 ± 12.31	138.97 ± 12.32	13.270	<0.001
	Serum albumin (g / L)	36.75 ± 2.31	40.10 ± 4.25	6.414	<0.001
	Fasting blood glucose (mmol/L)	5.76 ± 1.12	5.69 ± 1.10	0.422	0.674
	Blood uric acid (μ mol/L)	282.32 ± 30.19	299.08 ± 24.31	4.124	<0.001
	Blood creatinine (μ mol/L)	56.74 ± 5.38	61.23 ± 7.58	4.511	<0.001
	Transaminase (U / L)	19.12 ± 1.43	19.23 ± 1.52	0.497	0.620
	ALT (U / L)	15.64 ± 1.56	15.70 ± 1.49	0.264	0.792
	Blood potassium (mmol/L)	4.12 ± 0.24	4.15 ± 0.37	0.633	0.527
	Blood sodium (mmol/L)	141.35 ± 15.64	141.26 ± 16.14	0.038	0.970
	Blood calcium (mmol/L)	2.23 ± 0.24	2.28 ± 0.31	1.195	0.234
	Blood phosphorus (mmol/L)	1.10 ± 0.13	1.12 ± 0.15	0.948	0.345
	Blood magnesium (mmol/L)	0.83 ± 0.11	0.85 ± 0.10	1.277	0.203

Data were expressed as number (%) or mean ± SD. ALT, Alanine aminotransferase.

Besides, the fracture group exhibited higher serum β-CTX (1.25 ± 0.27 ng/mL vs. 0.30 ± 0.08 ng/mL), PINP (88.76 ± 6.89 ng/mL vs. 51.89 ± 3.41 ng/mL), and ALP levels (105.53 ± 12.31 U/L vs. 68.94 ± 7.48 U/L) compared to the non-fracture subgroup (P < 0.001 for all). Meanwhile, lumbar spine (L_1-4_) and femoral neck BMD were significantly reduced in the fracture group (0.28 ± 0.03 g/cm² and 0.29 ± 0.03 g/cm² vs. 0.55 ± 0.07 g/cm² and 0.43 ± 0.04 g/cm², respectively; P < 0.001) (**Table 5**).

**Table 5:** Comparison of BMD and serum β-CTX, PINP and ALP levels between the fracture and non-fracture group.

Items	β-CTX (ng/mL)	PINP (ng/mL)	ALP (U/L)	Lumbar vertebra L_1~4_ BMD (g/cm^2^)	Femoral neck BMD (g/cm^2^)
Fracture group (n=83)	1.25 ± 0.27	88.76 ± 6.89	105.53 ± 12.31	0.28 ± 0.03	0.29 ± 0.03
Non-fracture group (n=97)	0.30 ± 0.08	51.89 ± 3.41	68.94 ± 7.48	0.55 ± 0.07	0.43 ± 0.04
*t*	33.010	46.480	24.470	32.660	26.200
*P*	<0.001	<0.001	<0.001	<0.001	<0.001

Data were expressed as mean ± SD. ALP, alkaline phosphatase; β-CTX: BMD, bone mineral density; β-CTX, β-C-terminal telopeptide of type I collagen; PINP, procollagen type I N-terminal propeptide.

### 3.4 Factors associated with secondary fractures in patients with osteoporosis

As demonstrated in **Table 6**, logistic regression identified age ≥60 years (OR = 3.897, 95% CI: 2.314–5.664), manual labor occupation (OR = 1.321, 95% CI: 1.011-2.453), elevated β-CTX (OR = 6.665, 95% CI: 4.532-7.895), PINP (OR = 5.643, 95% CI: 3.409-9.032), and ALP (OR = 4.532, 95% CI: 3.452-7.429) as independent predictors of secondary fractures among patients with osteoporosis (all P < 0.05). The logistic regression model demonstrated a good fit as indicated by the Hosmer-Lemeshow test (χ² = 7.24, P = 0.51). No significant multicollinearity was detected among the independent variables, with all VIF below 3.5 (mean VIF = 2.1) (**Supplementary Table S2**).

**Table 6:** Analysis of factors associated with secondary fractures in patients with osteoporosis.

Parameters	*β*	*SE*	*Wald*	*P*	*OR*	95% CI
Age	0.512	0.563	8.792	0.000	3.897	2.314~5.664
Manual labor	0.554	0.453	3.422	0.032	1.321	1.011~2.453
Use of calcium agent/ vitamin D	0.489	0.552	5.642	0.005	2.453	1.894~4.662
Hemoglobin	0.634	0.392	3.045	0.041	1.043	0.786~2.495
Serum albumin	0.523	0.471	6.113	0.004	2.336	1.023~4.553
Blood uric acid	0.554	0.583	3.241	0.029	3.034	1.893~5.647
Serum creatinine	0.438	0.662	4.553	0.013	3.561	2.314~5.667
β-CTX	0.612	0.592	13.421	0.000	6.665	4.532~7.895
PINP	0.598	0.619	12.234	0.000	5.643	3.409~9.032
ALP	0.645	0.452	10.032	0.000	4.532	3.452~7.429
Lumbar vertebra L_1~4_ BMD	0.412	0.441	9.088	0.000	4.310	2.224~5.664
Femoral neck BMD	0.324	0.482	9.000	0.000	4.124	2.312~6.745

ALP, alkaline phosphatase; β-CTX: BMD, bone mineral density; β-CTX, β-C-terminal telopeptide of type I collagen; PINP, procollagen type I N-terminal propeptide.

### 3.5 Predictive value of β -CTX, PINP, ALP, and BMD for secondary fractures in patients with osteoporosis

**Figure 2:**
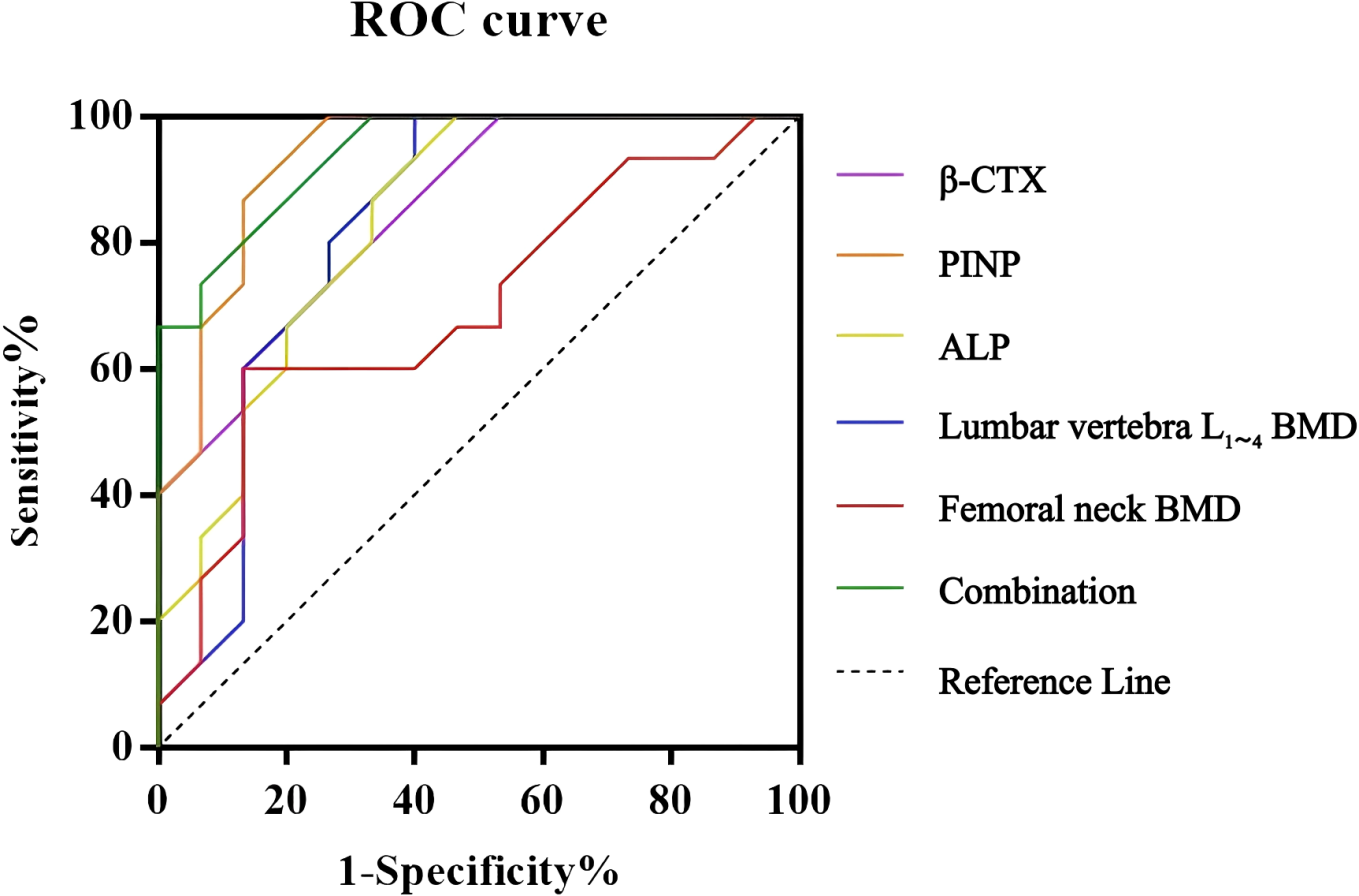
ROC curve analysis of β-CTX, PINP, ALP and BMD in predicting secondary fractures in patients with osteoporosis. ALP: alkaline phosphatase; β-CTX: BMD: bone mineral density; β-CTX: β-C-terminal telopeptide of type I collagen; PINP: procollagen type I N-terminal propeptide; ROC: receiver operating characteristics.

The ROC curve analysis showed that stand-alone serum β -CTX, PINP, and ALP demonstrated great predictive accuracy for secondary fractures, with all the AUC values exceeding 0.8.; among them, PINP had the highest performance (AUC = 0.879, 95% CI: 0.653-0.912, sensitivity = 85.40%, specificity = 83.62%). Of note, the combination of β-CTX, PINP, ALP, and BMD further improved the predictive efficacy, resulting in an outstanding AUC of 0.943 (95% CI: 0.795-0.997, sensitivity= 89.90%, specificity= 85.86%), outperforming individual markers. See **Table 7** and **Figure 2** for details.

**Table 7:** Predictive utility of β-CTX, PINP, ALP and BMD for secondary fractures in patients with osteoporosis.

Parameters	Optimal cutoff point	Sensitivity (%)	Specificity (%)	AUC	95%CI
β-CTX	0.97 ng/mL	83.42	81.90	0.851	0.714~0.900
PINP	73.42 ng/mL	85.40	83.62	0.879	0.653~0.912
ALP	87.95 U/L	82.11	80.83	0.830	0.681~0.898
Lumbar vertebra L_1~4_ BMD	0.39 g/cm^2^	78.65	72.32	0.745	0.676~0.792
Femoral neck BMD	0.33 g/cm^2^	76.75	75.64	0.712	0.623~0.786
Combination of these markers	—	89.90	85.86	0.943	0.795~0.997

AUC, area under the curve; ALP, alkaline phosphatase; BMD, bone mineral density; β-CTX, β-C-terminal telopeptide of type I collagen; PINP, procollagen type I N-terminal propeptide.

## 4 Discussion

The present study corroborates and extends current understanding of bone turnover markers (BTMs) in osteoporosis management by demonstrating significant elevations in serum β-CTX, PINP, and ALP levels in osteoporotic patients compared to healthy controls, alongside their inverse correlations with BMD and predictive utility for secondary fractures in patients with osteoporosis. These findings align with established pathophysiological mechanisms of osteoporosis, where accelerated bone remodeling disrupts the equilibrium between resorption and formation, leading to net bone loss and microarchitectural deterioration (1).

In osteoporosis, bone resorption increases while the degradation of type I collagen intensifies, leading to elevated blood levels of β-CTX, a collagen degradation product reflecting osteoclastic activity. When osteoclast activity rises, β-CTX levels surge, correlating with osteoporosis occurrence (9, 10). PINP, a marker of collagen synthesis linked to osteoblastic function, increases with higher type I collagen synthesis and is indicative of osteoblast activity (10, 11). Elevated ALP levels usually reflect increased osteoblastic activity and an active bone remodeling process, which are crucial for initiating and facilitating new bone mineralization and synthesis (12–14). The observed elevation in β-CTX, PINP and ALP levels underscores the imbalance between bone resorption and formation, consistent with the metabolic dysregulation driving osteoporosis progression (7). Our results are consistent with IOF recommendations recognizing β-CTX and PINP as reference markers for bone metabolism assessment (6). To minimize confounding effects of renal dysfunction on bone metabolism markers, patients with impaired kidney function (eGFR < 60 mL/min/1.73 m^2^) were excluded. This exclusion strengthens the validity of our findings, as CKD is known to independently alter β-CTX and ALP levels. Besides, negative correlations of serum β-CTX, PINP, and ALP levels with BMD at the lumbar spine (L1-4) and femoral neck were found, underscoring their potential for screening bone loss and assessing osteoporosis severity. Notably, our mixed-gender cohort reinforces the generalizability of these associations beyond postmenopausal populations.

Logistic regression delineates several independent factors that contribute to the risk of secondary fractures in patients with osteoporosis, including increased age, occupational type, use of calcium and vitamin D supplementation, and various biochemical markers such as hemoglobin, serum albumin, serum uric acid, and creatinine levels. Furthermore, β-CTX, PINP, ALP and BMD measurements at lumbar vertebrae L1–4 and the femoral neck, are also critical in this assessment. This underscores the multifactorial nature of fracture risk in osteoporotic patients and reinforces the necessity of a comprehensive evaluation of these parameters to accurately assess fracture vulnerability. For instance, manual labor occupations are likely to exacerbate mechanical stress on osteoporotic bones, while hypoalbuminemia may impair collagen synthesis and bone quality (11). Previous studies have indicated that bone metabolism disorders may be linked to increased fracture risk. Jiang and co-workers (15) found higher β-CTx and P1NP levels in patients with osteoporotic fracture. Jia and colleagues (16) demonstrated that elevated PINP is an independent risk factor for secondary fractures in postmenopausal women. Chen et al. (17) suggested that increased ALP indicates a higher risk of hip fractures. Consistently, the present study demonstrated that elevated levels of β-CTX, PINP, ALP are associated with the increased risk of secondary fractures among patients with osteoporosis and this might be due to greater osteoclast activity in fracture patients, leading to elevated β-CTX in circulation and increased synthesis of PINP and ALP to promote bone formation.

In this study, ROC curve was further used to analyze the prediction efficacy of β -CTX, PINP, ALP and BMD on secondary fractures in patients with osteoporosis. The results demonstrated stronger predictive performance of β -CTX, PINP and ALP for secondary fractures in patients with osteoporosis, relative to BMD. Notably, the study also highlighted the superior predictive efficacy of combined β -CTX, PINP, ALP with BMD for secondary fractures with an AUC> 0.9, outperforming individual markers, and also highlighted their complementary roles: while BMD quantifies cumulative bone loss, BTMs reflect metabolic activity driving skeletal fragility. In addition, their synergistic predictive value further supports integrating these biomarkers into fracture risk algorithms, such as FRAX.

The observed elevation in β-CTX, PINP, and ALP reflects a state of accelerated bone remodeling, driven by RANKL/RANK/OPG pathway activation and osteocyte dysfunction (18, 19). This imbalance leads to excessive osteoclastic resorption (evidenced by β-CTX release) and compensatory but insufficient bone formation (reflected by PINP/ALP), resulting in microarchitectural deterioration that compromises bone strength beyond BMD reduction (20, 21). Thus, our findings are biologically grounded in established pathways where high turnover directly increases fracture risk (22).

This present study has several limitations. First, its single-center, retrospective design with a relatively small sample size and a cohort of advanced age may introduce selection bias and limit the generalizability of the findings to younger osteoporosis populations or other geographic and healthcare settings. Second, although diurnal variations in BTMs were mitigated by standardized morning sampling, the lack of longitudinal measurements remains a shortcoming, as single-timepoint data could not capture temporal fluctuations in marker levels. Third, and most importantly, a key methodological shortcoming was the measurement of total ALP instead of the bone-specific isoform (BALP). Total ALP includes isoforms from the liver, kidney, and other tissues, which may confound its interpretation as a pure marker of bone formation. Although we excluded patients with known liver or renal dysfunction, this lack of specificity remains a significant limitation of our study that weakens the conclusions regarding the bone formation process. Further well-designed prospective studies are needed to address these limitations.

In conclusion, elevated serum β-CTX, PINP, and ALP levels in osteoporosis patients correlate inversely with BMD and independently predict secondary fracture risk. Combining β-CTX, PINP, and ALP with BMD enhances fracture risk stratification, advocating for their integration into clinical assessment tools. Future multicenter studies should validate these findings and address limitations.

## Data Availability

The original contributions presented in the study are included in the article/**Supplementary Material**. Further inquiries can be directed to the corresponding authors.
