# Supplementary material for: Bone turnover markers (β-CTX, PINP, ALP) in osteoporosis: correlation with bone loss and fracture risk stratification
Source: Front Endocrinol (Lausanne). 2026 Jan 5;16:1628434. doi: 10.3389/fendo.2025.1628434 (PMC12812736; doi:10.3389/fendo.2025.1628434)
Supplement: Supplementary file 1 [file DataSheet1.docx]

**Table S1. Comparison of lifestyle and nutritional characteristics between the fracture and non-fracture groups**

| Characteristic | Fracture group (n=83) | Non-fracture group (n=97) | Statistical value | *P*-value |
| --- | --- | --- | --- | --- |
| Smoking status, n (%) |  |  | χ² = 10.524 | 0.005 |
| Current smoker | 25 (30.1%) | 15 (15.5%) |  |  |
| Former smoker | 20 (24.1%) | 18 (18.6%) |  |  |
| Never smoker | 38 (45.8%) | 64 (65.9%) |  |  |
| Alcohol consumption, n (%) |  |  | χ² = 8.931 | 0.011 |
| ≥ 3 times/week | 22 (26.5%) | 12 (12.4%) |  |  |
| Occasional | 28 (33.7%) | 35 (36.1%) |  |  |
| Abstainer | 33 (39.8%) | 50 (51.5%) |  |  |
| Physical activity level, n (%) |  |  | χ² = 42.710 | <0.001 |
| Low | 18 (21.7%) | 55 (56.7%) |  |  |
| Moderate | 35 (42.2%) | 32 (33.0%) |  |  |
| High | 30 (36.1%) | 10 (10.3%) |  |  |
| Dietary calcium intake (mg/day), mean ± SD | 386.5 ± 105.7 | 488.2 ± 121.3 | t = 5.987 | <0.001 |
| Vitamin D supplementation, n (%) | 33 (39.8%) | 68 (70.1%) | χ² = 16.723 | <0.001 |

**Table S 2. Assessment of multicollinearity among the independent variables in the logistic regression model using VIF.**

| Independent Variable | VIF |
| --- | --- |
| Age | 1.32 |
| Manual labour | 1.87 |
| Use of calcium/vitamin D | 1.45 |
| Hemoglobin | 2.12 |
| Serum albumin | 2.08 |
| Blood uric acid | 1.93 |
| Serum creatinine | 2.35 |
| β-CTX | 2.98 |
| PINP | 3.21 |
| ALP | 3.45 |
| Lumbar spine BMD | 2.67 |
| Femoral neck BMD | 2.54 |
| Mean VIF | 2.1 |
